# Anion Exchange Impedes Subsequent Cation Exchange: Ion Mobility Is Altered by Vacancies and Ion Size

**DOI:** 10.1021/acs.inorgchem.4c04273

**Published:** 2025-01-10

**Authors:** Clarisse Doligon, Eli Rudman, Noah Ehrenberg, Cat Tuong Nguyen Dinh, Qi Luo, Katherine E. Plass

**Affiliations:** Department of Chemistry, Franklin & Marshall College, Lancaster, Pennsylvania 17601, United States

## Abstract

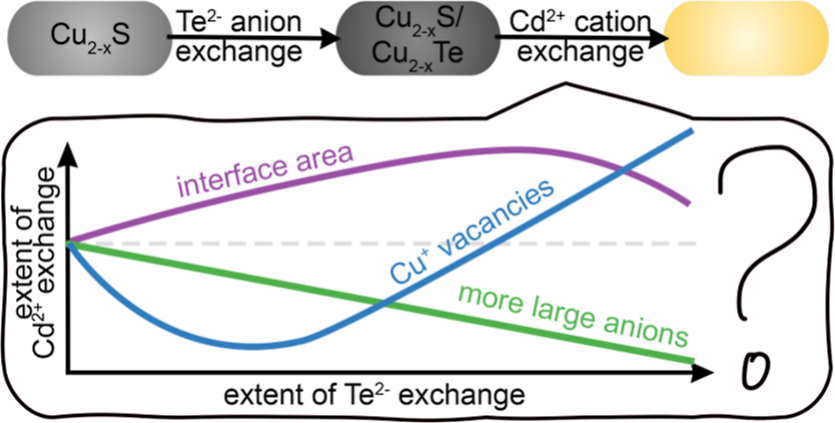

One method of achieving spatially specific, multi-component nanoheterostructures is to combine multiple forms of post-synthetic modification. Applying cation or anion exchange to Cu_2–*x*_S nanorods creates complex nanoheterostructures. Combining such anion and cation exchanges generates a system which uncovers the interplay between these two processes and understands the cooperativity between postsynthetic modifications more broadly. Cd^2+^ exchange was carried out on various plasmonic and nonplasmonic Cu_2–*x*_S/Cu_2–*x*_Te nanoheterostructures to test how the presence of Te^2–^ ions would affect the extent of Cd^2+^ incorporation. Three hypotheses were presented for how the presence of Cu_2–*x*_Te could alter the incorporation of Cd^2+^ and these were used to interpret the observed changes in the extent of Cd^2+^ exchange and crystalline phase of the resulting particles. We found that Te^2–^ anion exchange impedes subsequent Cd^2+^ cation exchange. Low extents of Te^2–^ exchange cause a phase change where ion mobility is slowed by a decrease in Cu^+^ vacancies. Higher extents of Te^2–^ exchange slow ion mobility due to the presence of large Te^2–^ ions.

## Abstract 2

Here, we asked how application of consecutive post-synthetic transformations of roxbyite Cu_2–*x*_S nanorods—Te^2−^ anion exchange followed by Cd^2+^ cation exchange—altered the rate of incorporation of Cd^2+^ ions. We proposed different hypotheses regarding how the presence of Te^2−^ may speed or slow a secondary exchange. Experiments revealed that Cd^2+^ ion movement is altered in Cu_2−*x*_S/Cu_2−*x*_Te structures by the changing Cu^+^ vacancy concentrations and impeded movement by large Te^2−^ ions.

## Introduction

Post-synthetic transformations (PSTs) of nanoparticles allow creation of complex phases and structures of nanomaterials that are otherwise difficult to directly synthesize, and a combination of PST can amplify structural complexity and give rise to tailored properties for optoelectronic, catalysis, and energy storage applications. Complicated heterostructures in nanomaterials have been made accessible through consecutive and simultaneous PST. Cation exchange allows replacement of the existing cations with new ones. Typically, the shape and crystal sublattice of the original particle are retained. Successive cation exchanges are a design framework to engineer desired nanomaterial heterostructures based on interface reactivity and crystal lattice compatibility. For example, numerous consecutive cation exchanges result in a megalibrary of multicomponent nanorods with controlled composition and placement.^1,2^ Consecutive cation exchange with or without etching creates new compositions and shapes.^3−5^

**Figure 1:**
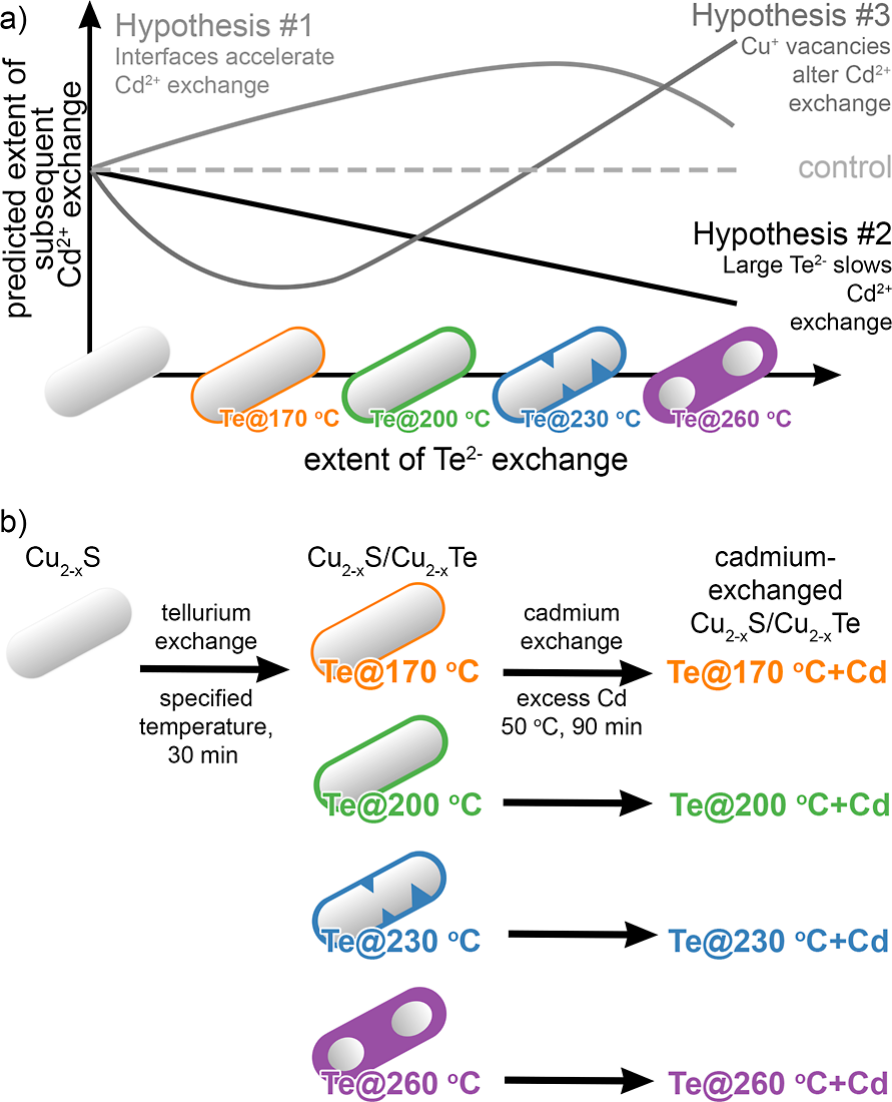
(a) Qualitative depiction of three hypothesized relationships between the extent of Te^2–^ exchange and the extent of subsequent Cd^2+^ exchange. (b) Schematic depiction of the conversion of Cu_2–*x*_S nanorods by consecutive Te^2–^ and Cd^2+^ exchange PSTs with the naming scheme based on the temperature of Te^2–^ exchange.

The process of anion exchange is more difficult than cation exchange because the larger size of anions hinders their incorporation and mobility. Recently, Te^2–^ exchange on Cu_2–*x*_S^6,7^ and Cu_2–*x*_Se^8^ have been used to create new forms of structural complexity. A Cu_2–*x*_Se_*y*_Te_1–*y*_ solid solution is formed from Cu_2–*x*_Se. Te^2–^ exchange on roxbyite Cu_2–*x*_S nanorods creates several different Cu_2–*x*_S/Cu_2–*x*_Te heterostructures depending on the extent of exchange (Figure 1a). Low levels of exchange result in a core–shell structure and a phase change to the nonplasmonic α-chalcocite phase.^7^ Higher levels of tellurium exchange form Cu_2–*x*_S domains within Cu_2–*x*_Te, first disordered and then a double-core structure. Te^2–^ exchange also offers an additional design element that can be combined with other PST transformations to create new structures. Copper sulfides,^1,2^ and tellurides,^9,10^ readily undergo cation exchange, leading us to ask how cation exchange would proceed on Cu_2–*x*_S/Cu_2–*x*_Te heterostructures. Multicomponent metal telluride heterostructures could have applications in photovoltaics, photocatalysis, up-conversion, thermoelectrics, or ion-storage batteries.^11^

Consecutive and simultaneous PSTs show patterns in reactivity that expose design rules important for the rational design of nanoheterostructures. Cation exchange is accelerated when there is lattice matching of the host crystal structure and the structure formed. This allows generation of different regioselectivities based on the shape and cation,^12^ numerous isomeric heterostructures,^1,13^ and leads to selectivity between cation exchange and metal deposition.^14,15^ Cation exchange of Cu_2–*x*_Se/Cu_2–*x*_S dot-in-rod structures shows that new cations sample the whole particle and select the more thermodynamically favorable selenide phases.^5^ Both disordered interfaces^2^ and Cu^+^ vacancies^16^ create reactive sites for subsequent cation exchange that accelerate incorporation of guest ions, even while ordered interfaces create kinetic barriers to exchange.^17^ Guest ion size and coordination alter the rate of diffusion and impact heterostructure selection in Cu_2–*x*_Te. Smaller, 4-coordinate Cd^2+^ and Hg^2+^ ions both diffuse more rapidly than larger, 6-coordinate Pb^2+^ and Sn^2+^ despite the fact that Hg^2+^ would have the most favorable hard–soft acid–base interaction with Te^2–^, while Cd^2+^ would have the least favorable.^9^

Based on foundational studies of the factors that alter the rate of cation exchange, we can present and test conflicting arguments for how an initial Te^2–^ exchange on Cu_2–*x*_S rods might affect the kinetics of subsequent cation exchange. Three simultaneous changes occur with Te^2–^ anion exchange that may change the rapidity of ion diffusion within the particles and thereby alter the facility of further exchange, leading to three competing hypotheses (Figure 1a). Hypothesis #1: Formation of Cu_2–*x*_S/Cu_2–*x*_Te heterostructures introduces disordered domain interfaces, which are active spots for cation exchange. This would predict that Cu_2–*x*_S/Cu_2–*x*_Te heterostructures would be more reactive toward cation exchange than pure Cu_2–*x*_S. Hypothesis #2: The larger size of the Te^2–^ ions slows the ion mobility into and out of the crystal structure. This would hinder cation exchange of Cu_2–*x*_S/Cu_2–*x*_Te heterostructures compared to pure Cu_2–*x*_S. The greater the Te^2–^ incorporation, the slower the cation exchange would progress. Hypothesis #3: The Cu^+^ vacancy levels alter the propensity for cation exchange. Increasing Cu deficiency creates Cu^+^ vacancies that facilitate ion diffusion and thus cation exchange.^16,18^ Te^2–^ anion exchange has two competing effects on the Cu deficiency of the crystals.^7^ At low levels of anion exchange, the copper deficiency in the center of the rod is decreased; at high levels of anion exchange, the copper deficiency increases until the stoichiometry is CuTe.^6,7^ If this hypothesis is accurate, we would predict that the rate of cation exchange would be lowest for Cu_2–*x*_S/Cu_2–*x*_Te heterostructures with the lowest vacancy levels (those that show LSPR quenching) and then increase as more Te^2–^ and more vacancies are present. Note that these hypotheses focus on factors that have previously been demonstrated to alter the rates of ion diffusion within particles. Various thermodynamic considerations, including bond dissociation energies, HSAB factors, and solubility products (*K*_sp_), have additional impacts on which ion exchanges are successful under what conditions, phase selectivity, and heterostructure formation.^19^

To identify the most valid hypothesis, we examined the extent of Cd^2+^ ion incorporation by cation exchange into a series of Cu_2–*x*_S/Cu_2–*x*_Te heterostructures (Figure 1b). We started with the first generation of Cu_2–*x*_S nanorods. Te^2–^ exchange was carried out (30 min of Te = TOP complex exposure at 170, 200, 230, and 260 °C) to create a second generation of particles termed Te@170 °C, Te@200 °C, Te@230 °C, and Te@260 °C, respectively. These Cu_2–*x*_S/Cu_2–*x*_Te heterostructures were subject to a relatively gentle Cd^2+^ cation exchange with an excess of Cd^2+^ to create a third generation of nanorods referred to as Te@170 °C + Cd, Te@200 °C + Cd, Te@230 °C + Cd, and Te@260 °C + Cd, depending on the temperature of the Te^2–^ exchange.^20^ The same Cd^2+^ exchange conditions were applied to Cu_2–*x*_S particles to serve as a baseline for comparing the extent of Cd incorporation. The Te^2–^ exchange temperatures and times were selected to sample the range of copper-deficiency levels, including low-level extents to see the effect of LSPR quenching and high-level extents approaching the 1:1 Cu:Te ratio. Cu_2–*x*_S, Cu_2–*x*_Se, and Cu_2–*x*_Te readily undergo cation exchanges with a variety of ions.^2^^21−23^ This general facility toward cation exchange required use of gentle temperature conditions to ensure partial Cd^2+^ exchanges so that differences in extent of incorporation would be apparent.

## Results and Discussion

**Figure 2:**
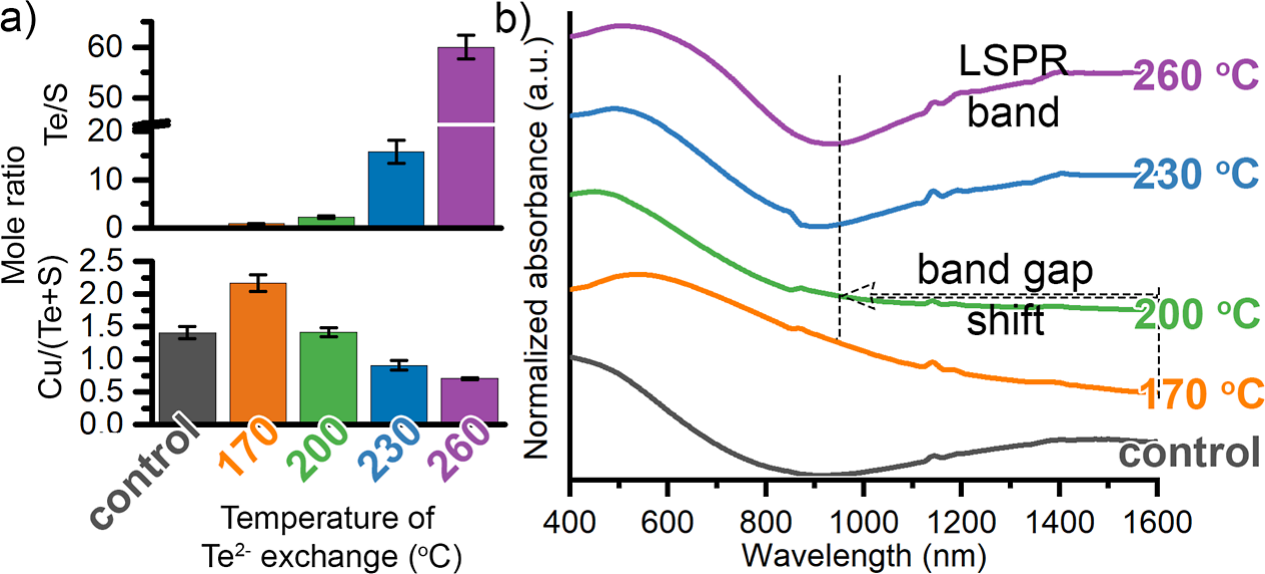
(a) SEM–EDS data showing the increase in the Te/S mole ratio with increasing temperature of Te^2–^ exchange (note the break in the *y*-axis that emphasizes the smaller ratios). Energy-dispersive spectroscopy (EDS) data also shows that the cation-to-anion ratio initially increases and then decreases. (b) UV/visible/NIR absorption spectra show first quenching of the LSPR (170 and 200 °C) then a return (230 and 260 °C) consistent with an initial decrease in Cu^+^ vacancy concentration.

**Figure 3:**
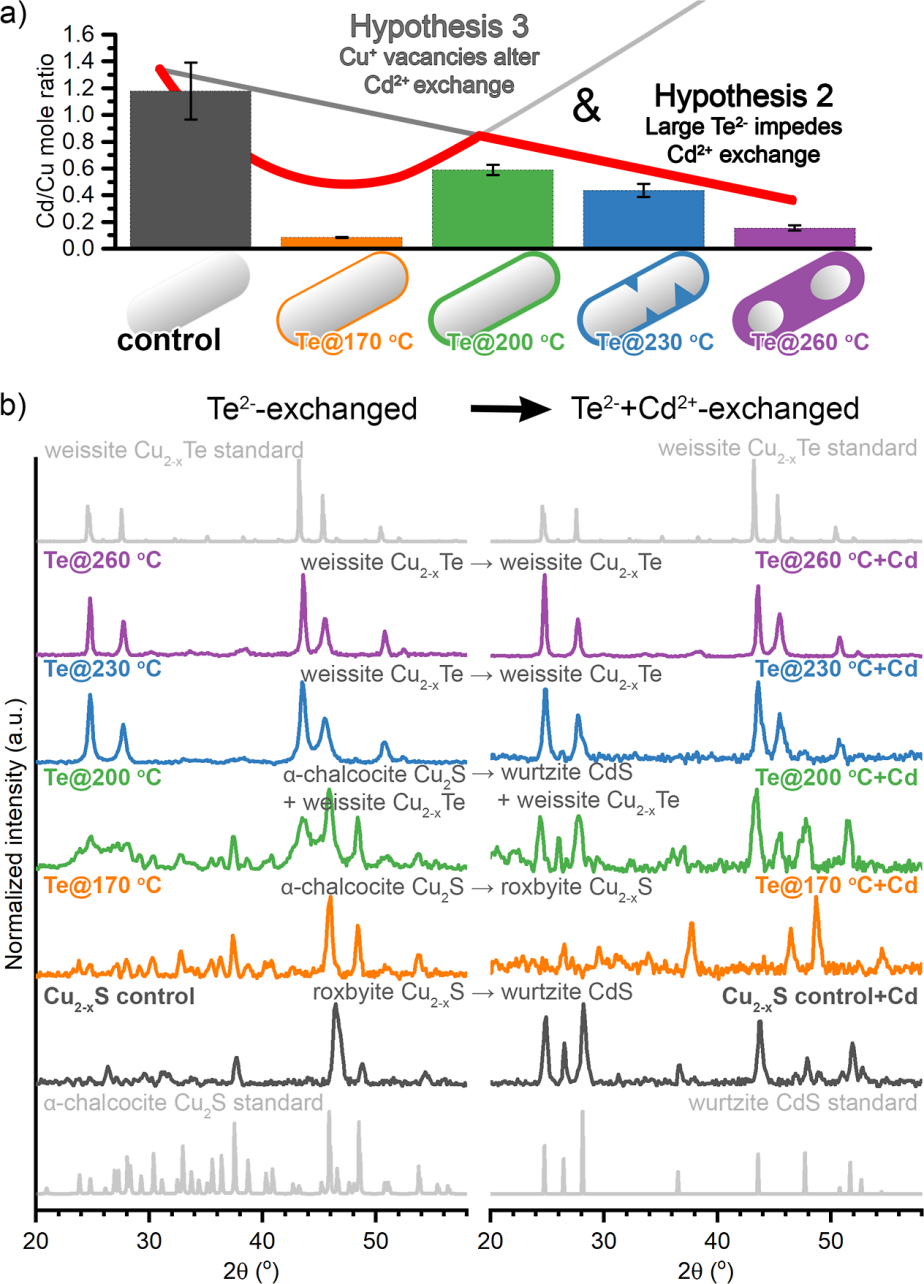
(a) Cd/Cu mole ratio of Cd^2+^-exchanged Cu_2–*x*_S/Cu_2–*x*_Te heterostructures, measured by SEM–EDS. Overlaid are the schematic representations of trends predicted if the Cu^+^ vacancy levels determined the rate of incorporation (Hypothesis #3) and if the size of the Te^2–^ ion slowed incorporation (Hypothesis #2). The red line shows the combination of these hypotheses consistent with the data. (b) PXRD showing the crystal structure of the Te^2–^-exchanged and Cd^2+^-exchanged nanorods, matched against roxbyite (ICDD 00-023-0958), α-chalcocite (ICDD 00-023-0961), weissite (ICDD 98-004-2156), and wurtzite (ICSD-31074).

By varying the temperature of Te^2–^ exchange, Cu_2–*x*_S/Cu_2–*x*_Te nanorods were obtained with crystal phases, heterostructures, composition, and optical properties (Figures 2, 3b, S1, and S2) that are consistent with previous reports and form a valid base for distinguishing Hypotheses #1, #2, and #3.^6,7^ The Cu_2-*x*_S/Cu_2–*x*_Te heterostructures vary from core–shell (Te@170 °C and Te@200 °C), irregular core–shell (Te@230 °C), and double core (Te@260 °C) (Figure S1), as needed to test Hypothesis #1. Te^2–^ incorporation increased with the temperature of the exchange reaction from a Te/S mole ratio of 0.9 ± 0.1 for Te@170 °C to 60 ± 2 for Te@260 °C (Figure 2a), as needed to test Hypothesis #2. Compared to the Cu_2–*x*_S nanorods, the Te@170 °C nanorods are less copper deficient, then the Cu^+^ vacancies continually increase for the Te@200 °C, Te@230 °C, and Te@260 °C nanorods, as needed to test Hypothesis #3. The cation-to-anion mole ratio is greatest for the Te@170 °C nanorods; the cation-to-anion ratio continually decreases from Te@170 to Te@260 °C as the temperature of Te^2–^ exchange increases (Figure 2a). These higher-temperature Te^2–^ exchanges result in the extraction of Cu^+^ ions likely by the trioctylphosphine. While trioctylphosphine typically etches Cu_2–*x*_S in the presence of oxygen,^3^ it can also extract Cu^+^ from intact particles.^24^ This behavior may be enabled by destabilization of the crystal lattice during anion exchange. At 170 °C, the thin Cu_2–*x*_Te shell promotes reorganization from the copper-deficient roxbyite Cu_2–*x*_S phase to the stoichiometric α-chalcocite Cu_2_S phase (Figures 3b and S2).^7^ This phase transformation promotes quenching of LSPR absorption. The Cu_2–*x*_S nanorods have a plasmon absorbance extending from 1600 to 1000 nm, while the Te@170 °C nanorods show suppression of this LSPR band and a shift in the band gap onset from 1000 nm for Te@170 °C out to 1600 nm. This is consistent with a Mossec-Burstein^25^ effect as empty states at the conduction band edge are filled, reducing the free carriers, red-shifting the LSPR band, and shrinking the band gap.^26,27^ Consistent with the lower cation-to-anion ratio, the absorbance spectrum of Te@200 °C nanorods shows more vacancies than the Te@170 °C nanorods (Figure 2a). The Te@200 °C nanorods do not have a prominent LSPR absorbance but the band gap onset blue shifts compared to the Te@170 °C nanorods indicating an increase in Cu^+^ vacancies, consistent with previous reports.^7^ Powder X-ray diffraction (PXRD) of Te@200 °C nanorods shows that they exhibit α-chalcocite Cu_2_S and weissite Cu_2–*x*_Te phases (Figure 3b). The weissite phase indicates that the transformation from the copper sulfide occurred with retention of the quasi-hexagonally close-packed anion sublattice.^6^ The Te@230 and Te@260 °C nanorods incorporate sufficient tellurium so that weissite Cu_2–*x*_Te is the only crystalline phase (Figures 3b and S2) and the LSPR absorption returns (Figure 2b).

Both control roxbyite Cu_2–*x*_S and the Cu_2–*x*_S/Cu_2–*x*_Te nanorods were exposed to excess Cd^2+^ under conditions to cause partial exchange of Cu_2–*x*_S (50 °C for 90 min) (Figure 1a). Partial cation exchange did occur for all Te^2–^-exchanged particles, resulting in particles with Cd/Cu mole ratios greater than 0.05 ± 0.03, a low but measurable value (Figure 3a). Transmission electron microscopy (TEM) shows the retention of the rod morphology consistent with cation exchange (Figure S1). Similar retention of particle morphology has been observed upon consecutive anion and cation exchange of ZnO tetrapods and CdO nanospheres^28^ with the difference that Kirkendall void formation is not observed in this system. This shape retention upon a second PST occurred despite the fact that anion exchange introduces stacking faults^6^ that might destabilize the particles.

The presence of Te^2–^ in the Cu_2–*x*_S/Cu_2–*x*_Te nanorods inhibits incorporation of Cd^2+^ into these nanorods, refuting Hypothesis #1. While measurable Cd^2+^ incorporation is observed in all resultant rods (Figure 3a and Table S2), EDS of the Cd^2+^-exchanged nanorods shows much lower Cd/Cu ratios compared with the control where Cd^2+^ exchange was carried out on Cu_2–*x*_S nanorods. The crystalline phases of the Cd^2+^ exchanged nanorods are consistent with this EDS data (Figures 3b and S2). The Cd^2+^-exchanged nanorod control showed conversion to the wurtzite CdS as expected due to retention of the pseudohexagonally close-packed sulfide ions. None of the nanorods that experienced both Te^2–^ and Cd^2+^ exchange showed phase-pure wurtzite CdS. Despite the Cu_2–*x*_S/Cu_2–*x*_Te interfaces that may have created reactive sites that could accelerate cation exchange, such acceleration was not observed thereby ruling out Hypothesis #1 (Figure 1b). This may be because the interfaces do not extend to the surface or because the transport of Cd^2+^ is occurring along planes perpendicular to the transport of Te^2–^.

For Cu_2–*x*_S/Cu_2–*x*_Te nanorods with the lowest Cu^+^ vacancy concentration, Cd^2+^ incorporation is most hindered; this is consistent with Hypothesis #3. As discussed above, Te@170 °C was the least Cu-deficient Te^2–^-exchanged nanorods. Cd^2+^ exchange on the pure Cu_2–*x*_S nanorods resulted in a Cd:Cu ratio of 1.2 ± 0.2, and for Te@170 °C + Cd, this decreased to 0.085 ± 0.003. Consistent with this very low Cd incorporation, the Te@170 °C + Cd nanorods retain a roxybite Cu_2–*x*_S copper sulfide phase. Also consistent with Hypothesis #3, the Cd incorporation increases between Te@170 °C + Cd and Te@200 °C + Cd. Te@200 °C particles had a higher Cu^+^ vacancy concentration than Te@170 °C as indicated by the band gap shift and the Cu/(Te + S) mole ratio. The Cd/Cu ratio for the resultant Te@200 °C + Cd particles increased to 0.59 ± 0.4, indicating that Cd^2+^ exchange is facilitated.

If the Cu^+^ vacancies were the only factor determining the rate of Cd incorporation into Cu_2–*x*_S/Cu_2–*x*_Te particles (Hypothesis #3), the Cd/Cu ratio should steadily increase from Te@200 °C to Te@260 °C to Cu, but that is not what we observe. Despite the prominent LSPR and increasing Cu^+^ vacancy concentrations for Te@230 °C + Cd (Figure 2b) and Te@260 °C + Cd,^7^ the Cd/Cu mole ratio drops between Te@200 °C + Cd (0.59 ± 0.04) and Te@230 °C + Cd (0.44 ± 0.04) and drops further for Te@260 °C + Cd (0.05 ± 0.03) (Figure 3a and Table S2). Thus, the Te@200 °C + Cd nanorods have the greatest Cd/Cu mole ratio of all of the Cd^2+^-exchanged particles. The PXRD of Te@200 °C + Cd nanorods shows a mixture of wurtzite CdS and weissite Cu_2–*x*_Te (Figures 3b and S2). In comparison, the PXRD of Te@200 °C before Cd^2+^ exchange was a mixture of α-chalcocite Cu_2_S and weissite Cu_2–*x*_Te (Figures 3b and S2), suggesting that the Cu_2_S component converted to CdS but the Cu_2–*x*_Te phase resisted transformation. Above 200 °C, the Cd incorporation into Te@230 °C + Cd and Te@260 °C + Cd nanorods decreased with increasing tellurium incorporation, which is more consistent with Hypothesis #2. Given the low levels of Cd^2+^ exchange, the PXRD of the Te@230 °C + Cd and Te@260 °C nanorods maintained the initial weissite Cu_2–*x*_Te phase (Figures 3b and S2). Overall, this decrease in Cd incorporation shows that regardless of the potential for formation of various regioselectivities or interfaces, increasing the Cu_2–*x*_Te component within the core of the particle impedes cation exchange.

Maximum Cd incorporation occurred on Cu_2–*x*_S/Cu_2–*x*_Te nanorods with intermediate extents of Te incorporation, which is not consistent with any of the three hypotheses alone; instead, we need to consider that different factors dominate in different regimes. When Cu_2–*x*_Te is only present as a thin shell (Te@170 °C), the incoming Cd^2+^ ions are slowed with respect to baseline but they still penetrate quickly enough that the rate is limited by the availability of Cu^+^-vacancy sites to facilitate ion movement (Hypothesis #3). As Cu_2–*x*_Te penetrates the nanorod, the extent of Cd^2+^ incorporation is limited by the difficulty of transporting the large guest Cd^2+^ past the similarly large Te^2–^ ions, consistent with Hypothesis #2. Taken overall, the Te@200 °C and Te@230 °C particles have the optimal balance of enough Cu^+^ vacancies but not too much Te^2–^ to facilitate maximum Cd incorporation (Figure 3a). This new insight provides cues for designing nanoheterostructures with variable amounts of Cd.

**Figure 4:**
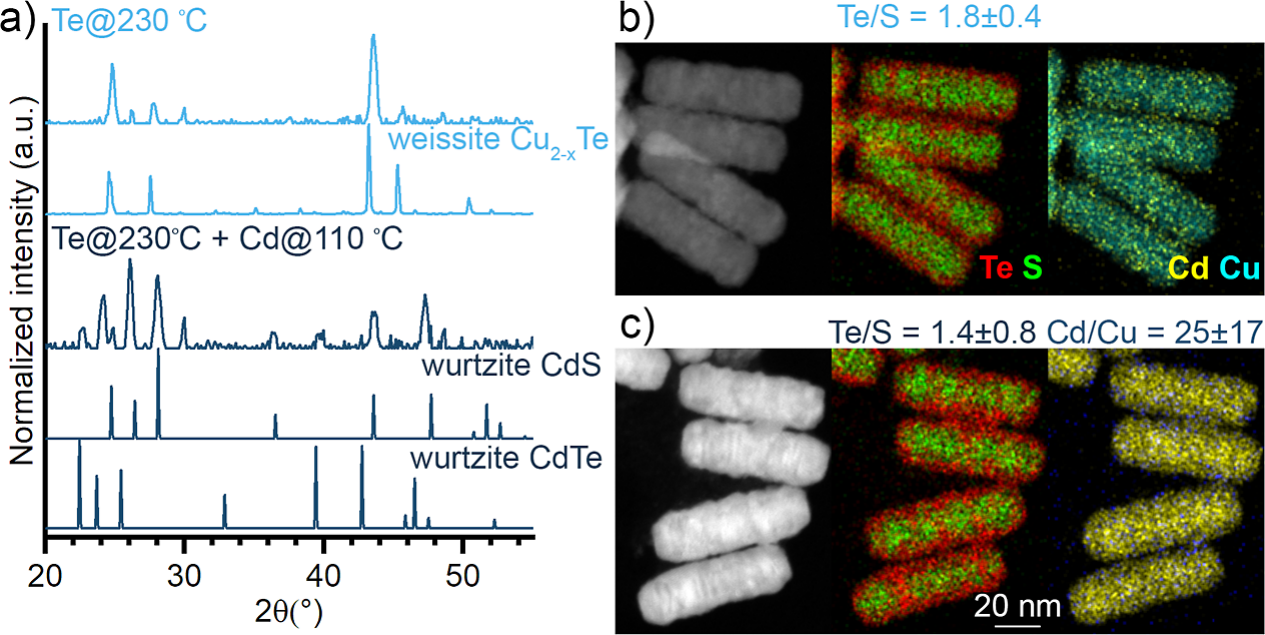
Composition of Cu_2–*x*_S nanorods after Te exchange for 30 min at 230 °C followed by Cd exchange at 110 °C for 90 min. (a) PXRD shows the crystal phase transforms from weissite Cu_2–*x*_Te (ICDD-98-004-2156) to a mixture of wurtzite CdS (ICSD-31074) and wurtzite CdTe (ICSD-620518). (b) STEM–EDS maps of the Te-exchanged rods show an irregular Cu_2–*x*_Te shell surrounding a Cu_2–*x*_S core. (c) After Cd exchange, the Cu_2–*x*_Te shell appears to have made in-roads into the Cu_2–*x*_S core but is largely intact while the Cu has been fully replaced by Cd (Cu is 2 ± 1 atomic %).

Combining different PSTs allows the formation of complicated heterostructures of metal chalcogenides. Using the design insight that the Cu_2–*x*_S/Cu_2–*x*_Te nanorods with intermediate amounts of Te^2–^ will be most amenable to Cd^2+^ cation exchange, we designed a new geometry of the CdS/CdTe nanorod. Te@230 °C nanorods were subject to a more aggressive Cd^2+^ exchange (110 °C, 90 min, Supporting Information). This combination of Te^2–^ and Cd^2+^ (Te@230 °C + Cd@110 °C) exchange results in the formation of a wurtzite CdS/CdTe core–shell heterostructure in which the hexagonal anion sublattice, shape, and original sulfide/telluride core–shell geometry are retained (Figures 4 and S4). The Te@230 °C nanorods showed an irregular outer shell of weissite Cu_2–*x*_Te (Figure 4a,b). STEM–EDS of Te@230 °C + Cd@110 °C shows that Cd^2+^ exchange at a higher temperature (110 °C) pushed incorporation of Cd^2+^ into both Cu_2–*x*_S and Cu_2–*x*_Te domains (Figures 4b, S3, and S4), whereas at 50 °C, only the Cu_2–*x*_S domain exchanged (Figure 3b). The resultant Cd/Cu mole ratio is 25 ± 17 indicating near-complete Cd^2+^ exchange. STEM–EDS maps show Cd throughout the Te@230 °C + Cd@110 °C particles, while Cu is present at background levels (Figures 4c, S3, and S4). PXRD shows a mixture of wurtzite CdTe and CdS, demonstrating retention of the hexagonal anion sublattice. The STEM–EDS maps of Te and S before (Figures 4b and S3) and after (Figures 4c, S3, and S4) Cd^2+^ exchange show that Te is on the outside of the particles, building up into irregular areas of greater Te concentration. Te penetrates further into the Cd^2+^-exchanged particles as though it was slightly mobilized by the Cd^2+^ movement but largely remains as a shell. This new wurtzite CdS/CdTe core/shell heterostructure adds to the existing variety of CdTe/CdS or CdS/CdTe core/shell particles,^29,30^ with new geometry and interfaces, and represents a new way to obtain CdTe-containing nanoheterostructures.

## Conclusions

We have demonstrated that a combination of PST, specifically anion and then cation exchange, is a powerful system in order to understand the kinetics of sequential PST and to design new heterostructures. Extension of this multigenerational PST approach can lead to an entire library of new heterostructures with mixed cations and anions, one of which was demonstrated here. The tunable creation of various Cu_2–*x*_S/Cu_2–*x*_Te heterostructures enabled a systematic evaluation of how factors that hinder and enable ion incorporation come into play for Cd^2+^ exchange. Specifically, we identified one regime in which a lack of Cu^+^ vacancies is the rate-limiting factor and a second regime in which the bulky size of the Te^2–^ ions is the limiting factor. While these two factors are sufficient to explain behavior here, additional factors may be in play here or in analogous systems and this general approach can help uncover them. Exploration of further variations of cation and anion exchanges could uncover the dominant factors affecting the kinetics of subsequent incorporation for ions of various sizes, charges, and lattice matches. Employing systems with different regioselectivities could better reveal the role of interfaces; perhaps ordered interfaces impede subsequent exchange, while disordered interfaces or interfaces with a certain crystallographic orientation accelerate it? Kinetic factors might combine with thermodynamic preferences to enable design of ever-more complex metastable, multicompound nanoheterostructures and new catalytic or optoelectronic functionality.

## Experimental Section

### Reaction Setup

The procedures employed a standard Schlenk line setup or an Ar gas manifold. A flame-dried three-neck round-bottom flask with a magnetic stir bar is connected to a reflux condenser. The condenser connects to the Schlenk line or to a mineral oil bubbler. The two open necks of the flask are sealed with silicone septa that have a needle connecting the Ar gas and a thermocouple. The temperature was controlled by heating mantles placed on magnetic stir plates.

### General Safety Concerns

The synthetic methods are performed under air-free conditions at elevated temperatures using high-boiling-point solvents. As such, care should be taken to ensure proper monitoring and handling. For example, burns have been reported from exposure to high-temperature oleylamine. The safety data sheets for all chemicals used in the reactions should be reviewed, and proper personal protective equipment should be used. These reactions should be performed in a properly functioning fume hood while wearing the appropriate personal protective equipment.

### Chemicals

The reagents used for the synthesis of Cu_2–*x*_S nanorods include copper nitrate trihydrate (≥99.9%), trioctylphosphine oxide (90%), 1-octadecene (90%), 1-dodecanethiol (98%), and *tert*-dodecanethiol (98.5%). Additional reagents used for tellurium anion exchange included tellurium (99.8%) and trioctylphosphine (97%). Cadmium cation exchange required cadmium acetate dihydrate (≥98%), oleylamine (70%), and dibenzyl ether (≥98%). Solvents used for washing particles included isopropyl alcohol, ethanol, acetone, toluene, and heptane. All reagents were obtained from Sigma-Aldrich.

### Synthesis of Cu_2–*x*_S Nanorods

Nanorods were synthesized as previously reported^6^ based on literature synthesis.^20^ Under Schlenk line conditions, Cu(NO_3_)_2_·3H_2_O (562 mg, 0.23 mmol), trioctylphosphine oxide (5.8 g, 1.5 mmol), octadecene (30 mL), and oleylamine (0.5 mL) were added to a 100 mL three-neck flask and placed under Ar flow. The mixture was degassed at 80 °C for 30 min, forming a blue solution. A mixture of *tert*-dodecanethiol (20 mL, 1.5 mmol) and dodecanethiol (2 mL, 0.0835 mmol) was separately degassed with Ar bubbling. After 30 min, the flask containing the copper precursor was cycled with Ar and vacuum three times, with each cycle lasting 5 min, then placed under an Ar blanket. The reaction temperature was increased to 180 °C within 5–10 min by placing the flask in a preheated heating mantle. At 130 °C, *tert*-dodecanethiol/1-dodecanethiol mixture (15 mL) was injected by a syringe to yield a green-/yellow-colored solution. When the temperature reached 180 °C, the solution became dark but not turbid, indicating that Cu_2–*x*_S nuclei formed. After approximately 5 min at 180–185 °C, the suspension became turbid. The flask was held at this temperature for 20–30 min after the observation of turbidity. After this growth time, the flask was cooled rapidly by removing the heating mantle and placing the flask into a room temperature water bath. When the temperature was at ∼40 °C, toluene (4 mL) was injected into the reaction mixture. Particles were precipitated with addition of isopropyl alcohol (40 mL) followed by centrifugation for 10 min at 6000 rpm. The particles were resuspended in hexane, precipitated with an equal volume of isopropyl alcohol and centrifuged twice to wash. The final brown product was resuspended in 10 mL of hexane.

### Tellurium Exchange

Tellurium exchange was carried out as previously reported.^6^ Te (0.038 g, 0.3 mmol), trioctylphosphine (1.2 mL, 0.269 mmol), and 1-octadecene (5 mL) were combined in a 25 mL 3-neck round-bottom flask. The mixture was degassed under Ar(g) for 20 min at 200 °C and then heated or cooled to the desired reaction temperature. The Cu_2–*x*_S nanorods (20 mg) were suspended in oleylamine in a septum-capped vial and purged under Ar(g) for 5 min. The vial was then sonicated for 5 min. The nanorods were swiftly injected into the flask and allowed to react for a desired reaction time. Here, we chose reaction temperatures of 170, 200, 230, and 260 °C for 30 min. The reaction mixture was then removed from heat and cooled using a water bath. The contents were transferred into a centrifuge tube and combined with ethanol (20 mL) and centrifuged for ten min at 6000 rpm. The particles were washed once more with heptane and ethanol.

### Cadmium Exchange

Cadmium exchange with an excess of Cd was carried out according to literature procedures.^20^ Cd(OAc)_2_·3H_2_O (0.300 g), oleylamine (8 mL), 1-octadecene (2 mL), and dibenzyl ether (15 mL) were combined in a 50 mL 3-neck round-bottom flask. The mixture was degassed under Ar(g) at 100 °C on a hot plate for 60 min, then cooled down to reaction temperature for injection of nanorods. The roxbyite nanorods (20 mg) were degassed under Ar(g) then suspended in trioctylphosphine (3 mL) with sonication for 45 min. The particles were swiftly injected into the flask containing a cadmium complex at either 50 or 100 °C for 90 min. Then, the reaction was then removed from heat and cooled using a water bath. The contents were transferred into a centrifuge tube, combined with isopropyl alcohol and centrifuged for 10 min at 6000 rpm. The particles were washed once more with isopropanol and hexane.

### Characterization

Powder X-ray Diffraction After the nanoparticles were cleaned and resuspended in heptane, they were cast onto glass slides and allowed to dry. The PXRD data were collected using a PANalytical X’Pert Pro X-ray diffractometer with Cu K_α_ radiation. The samples were scanned with 10 repetitions at a current of 40 mA and voltage of 45 kV. Using the PANalytical HighScore Plus software, the ten scans were summed and peaks were compared with patterns from the ICDD database to determine the structure of the nanoparticles. Crystal structure and powder diffraction simulations were performed using CrystalMaker and CrystalDiffract from CrystalMaker Software Ltd., Oxford, England.

### Transmission Electron Microscopy

Samples were prepared by placing a drop of nanoparticles suspended in toluene on a Au-supported Formvar carbon film 400 mesh TEM grid (Electron Microscopy Sciences). Low-resolution TEM images of the particles were obtained using a Delong Instruments LVEM25 Low-Voltage TEM at Franklin & Marshall College. The LVEM25 was operated under 25 kV with the Zyla 5.5 Scientific CMOS camera with appropriate alignments and enhancements. ImageJ software was used to analyze the TEM images.

### Scanning Electron Microscopy/Energy-Dispersive X-ray Spectroscopy

Nanoparticles previously cast onto the PXRD slides were immobilized on a small piece of conductive carbon tape and affixed to a metal stub. SEM and EDS of the sample were then carried out at 20 kV with an EvexMini-SEM. Atomic percents were measured in 6 different areas for each sample. The average and standard deviations of the ratios are reported in Figures 1, 3, 4, and Table S2.

### HAADF STEM/EDS Mapping

Samples were prepared by placing a drop of nanoparticles suspended in toluene on a Au-supported Formvar carbon film 400 mesh TEM grid (Electron Microscopy Sciences). The microscope employed was an FEI Talos F200X with a SuperX EDS at 200 kV in the Materials Characterization Laboratory at Pennsylvania State University. ImageJ software was used to analyze the HR-TEM images. Velox software was used to interpret the STEM–EDS element map data.

## Supporting Information Available

The following files are available free of charge. The Supporting Information is available free of charge at https://pubs.acs.org/doi/10.1021/acs.inorgchem.4c04273.

Details of author contributions, information about data repository, table of EDS values, TEM images of Te^2–^-exchanged and Cd^2+^-exchanged particles, additional PXRD comparisons, and additional STEM–EDS images of the fully Cd^2+^-exchanged particles (PDF)
