# Supplementary material for: Anion Exchange Impedes Subsequent Cation Exchange: Ion Mobility Is Altered by Vacancies and Ion Size
Source: Inorg Chem. 2025 Jan 10;64(2):978–85. doi: 10.1021/acs.inorgchem.4c04273 (PMC11752502; doi:10.1021/acs.inorgchem.4c04273)
Supplement: Supplementary file 1 — ic4c04273_si_001.pdf [file ic4c04273_si_001.pdf]

SUPPORTING INFORMATION

Anion exchange impedes subsequent cation exchange: Ion mobility is altered by vacancies and ion size

*Clarisse Doligon, Eli Rudman, Noah Ehrenberg, Cat Tuong Nguyen Dinh, Qi Luo, and Katherine E. Plass**

Department of Chemistry, Franklin & Marshall College, Lancaster PA 17601, USA

*kplass@fandm.edu

Table of Contents	Page
**Data repository information**	**S2**
**Table S1.** Author contributions according to CRediT Contribution Roles Taxonomy	**S2**
**Table S2.** Summary of SEM-EDS quantification as presented in Figures 2, 3, and 4.	**S3**
**Figure S1.** HAADF images and S/Te EDS maps of particles Te^{2-} exchanged at 170, 200, 230, and 260 °C.	**S4**
**Description of STEM-EDS maps**	**S5**
**Figure S2.** PXRD patterns of $Cu_{2-x}S$ nanorods after Te^{2-} exchange (left) and consecutive Cd^{2+} exchange (right) with matching database patterns.	**S6**
**Figure S3.** Individual STEM-EDS maps of Te@230 °C particles before (top) and after (bottom) Cd-exchange at 110 °C to create new wurtzite CdS/CdTe nanoheterostructures.	**S8**
**Figure S4.** Line scans of a STEM-EDS map of Te@230 °C+Cd@110 °C particles showing the irregular CdS/CdTe core/shell structure.	**S9**

Data repository information

Raw data used to produce figures in this manuscript can be found at Doligon, C.; Rudman, E.; Ehrenberg, N.; Nguyen Dinh, T.C.; Luo, Q.; Plass, K. E. *Open Science Framework Data repository*. https://osf.io/vq7a2/.

Table S1. Author contributions according to CRediT Contribution Roles Taxonomy

**Author**	**Contribution**	**Detail**
Clarisse Doligon	Investigation, Visualization, Writing – Original Draft.	Designed Figure 1, drafts of Figures 2, 3, and 4. Carried out experiments shown in Figures 2, 3, and 4.
Eli Rudman	Investigation, Writing – Review & Editing	Carried out experiments shown in Figures 2 and 3.
Noah Ehrenberg	Investigation, Visualization, Writing – Review & Editing	Carried out experiments shown in Figures 2 and 3. Created Figure S2.
Cat Tuong Nguyen Dinh	Investigation, Verification, Writing – Review & Editing	Carried out experiments shown in Figures 2 and 3.
Qi Luo	Conceptualization, Methodology, Investigation	Carried out preliminary experiments that demonstrated multiple post-synthetic transformations. Developed conceptual approach to understanding various aspects of sequential Cd^{2+} and Te^{2-} exchanges.
Katherine E. Plass	Conceptualization, Resources, Writing – Original Draft, Supervision, Project administration, Funding acquisition	Initiated, oversaw, and acquired funding for experiments. Wrote manuscript and finalized figures.

Table S2. Summary of SEM-EDS quantification as presented in Figures 2, 3, and 4.

Measurements were taken in six different areas for each sample to determine the average and standard deviation.

After Te^{2-} exchange			After partial Cd^{2+} exchange		
	Te/S mole ratio	Cu/(Te+S) mole ratio		Te/S mole ratio	Cd/Cu mole ratio
$Cu_{2-x}S$ control	na	1.40±0.09		na	1.2±0.6
Te@170 °C	0.87±0.01	2.2±0.1	Te@170 °C+Cd	0.21±0.01	0.085±0.003
Te@200 °C	2.2±0.3	1.42±0.07	Te@200 °C+Cd	0.46±0.01	0.58±0.04
Te@230 °C	16±2	0.91±0.07	Te@230 °C+Cd	3.3±0.4	0.44±0.05
Te@260 °C	60±2	0.70±0.02	Te@260 °C+Cd	16±3	0.05±0.02
			After full Cd^{2+} exchange		
Te@230 °C	1.8±0.4	1.4±0.1	Te@230 °C+Cd@110 °C	1.4±0.8	25±17

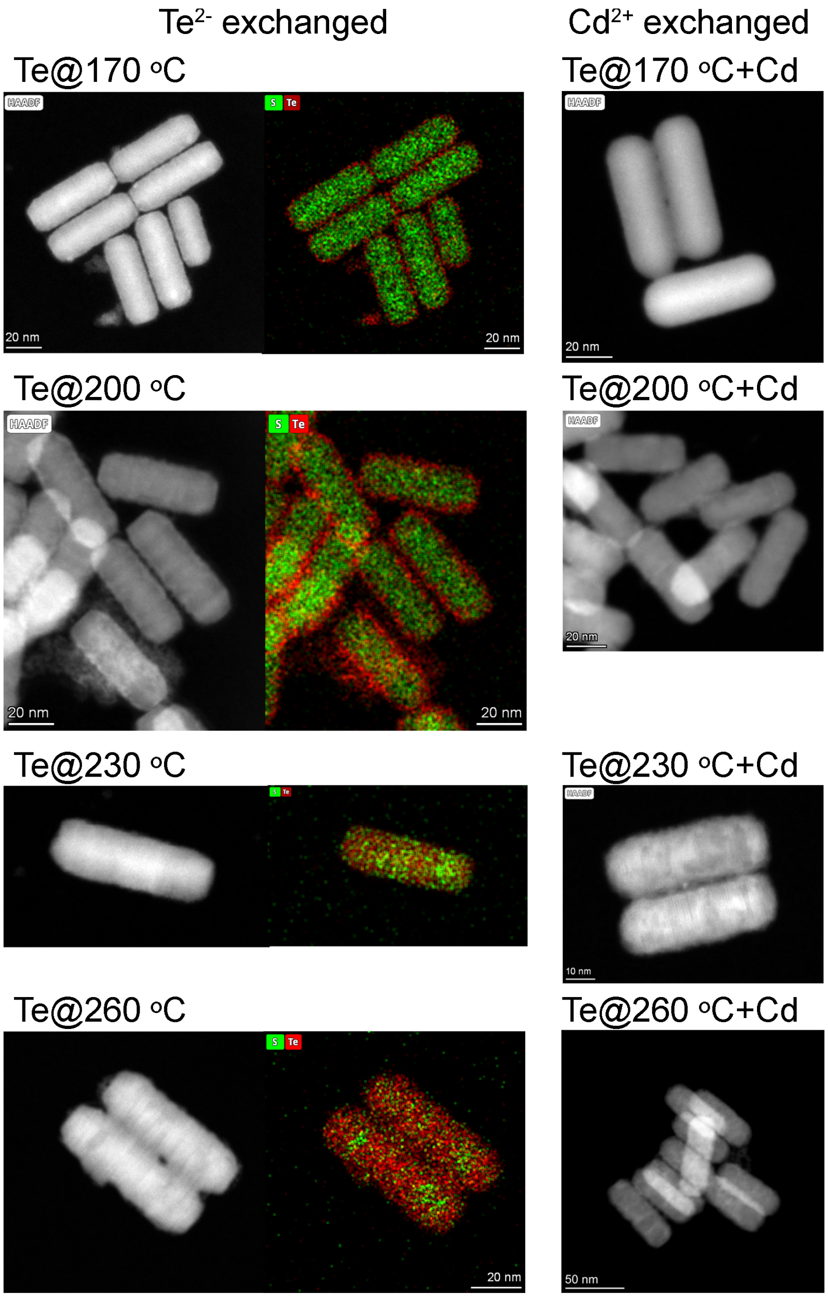

Figure S1. HAADF images and S/Te EDS maps of particles Te^{2-} exchanged at 170, 200, 230, and 260 °C, verifying the expected retention of the nanorod morphology and the expected regioselectivity of as previously published.[1,2] HAADF or TEM images of these $Cu_{2-x}S/Cu_{2-x}Te$ particles after Cd^{2+} exchange demonstrating that rod morphology is maintained ever after 2 post-

synthetic transformations, though particles get clumpy after Cd^{2+} exchange at higher temperatures.

STEM-EDS mapping description

The HRTEM and STEM-EDS of $Cu_{2-x}S/Cu_{2-x}Te$ particles has previously been reported,[1,2] and the STEM-EDS maps of the specific samples presented here generally show the same evolution of nanoheterostructure. Te@170 °C particles show only a thin layer of Te surrounding $Cu_{2-x}S$ rods, which gets thicker for Te@200 °C particles. The $Cu_{2-x}Te$ shells are of slightly variable thicknesses, indicating that these interfaces are points of disorder that could promote cation exchange. HRTEM[1] of similar core-shell particles shows that the shell consists of different crystalline domains with boundaries at the $Cu_{2-x}S/Cu_{2-x}Te$ interface. Te@230 °C particles have a more irregular structure with $Cu_{2-x}Te$ areas that penetrate into the particle. This creates further disordered interfaces that could promote subsequent cation exchange. Te@260 °C particles show areas where $Cu_{2-x}S$ cores are present within a primarily $Cu_{2-x}Te$ rod.

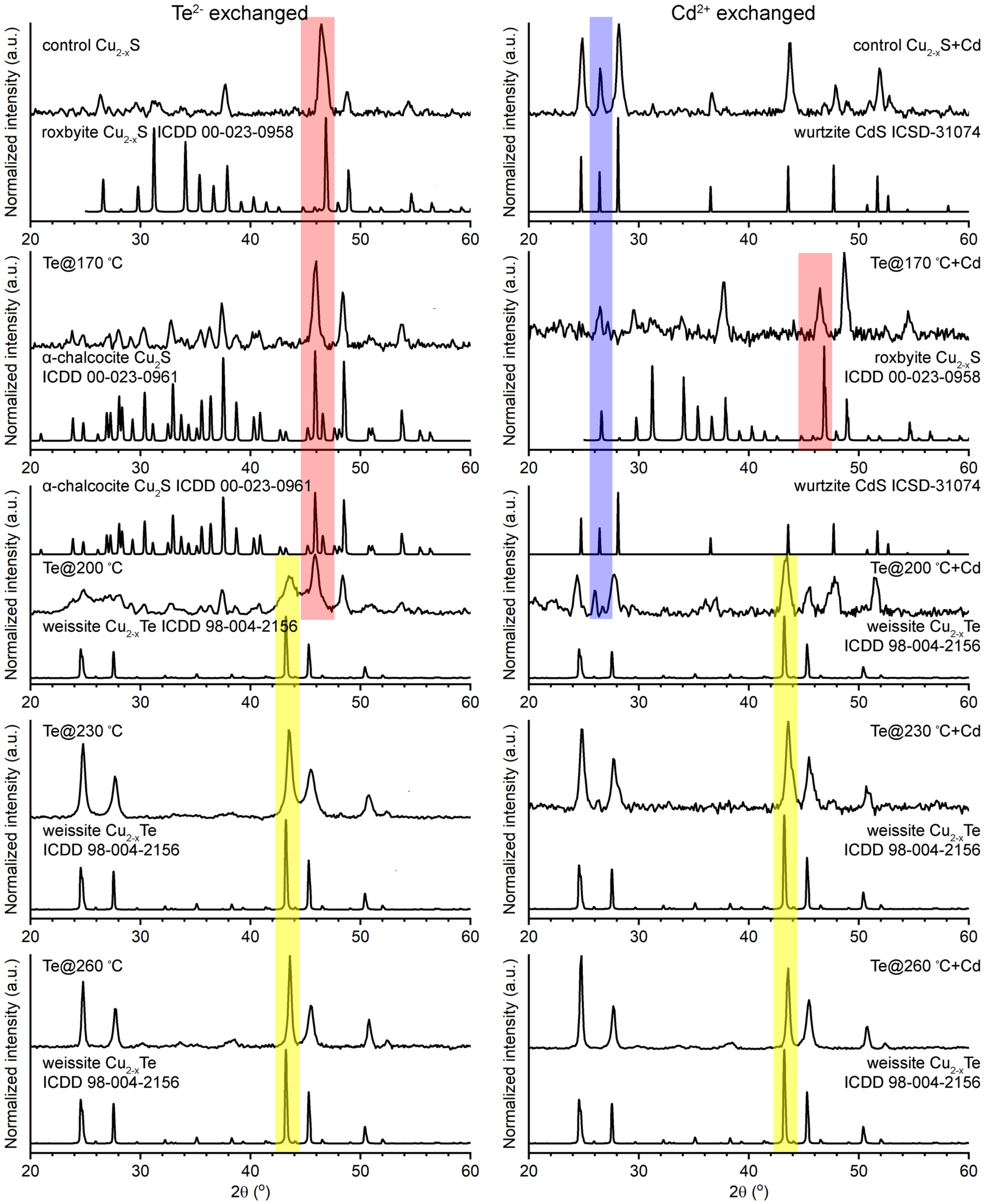

Figure S2. PXRD patterns of $Cu_{2-x}S$ nanorods after Te^{2-} exchange (left) and consecutive Cd^{2+} exchange (right) with matching database patterns. Key peaks in each structure have been highlighted. The red areas show the most prominent diffraction peak for $Cu_{2-x}S$, including the

shift from 47 °2θ to 46 °2θ indicative of a shift from copper-deficient roxybite to stoichiometric α-chalcocite. The most prominent weissite $Cu_{2-x}Te$ diffraction peak is highlighted in yellow. Though weissite $Cu_{2-x}Te$ and wurtzite CdS have many very similar peak positions, the appearance of diffraction at 26 °2θ (blue) is indicative of the wurtzite CdS structure.

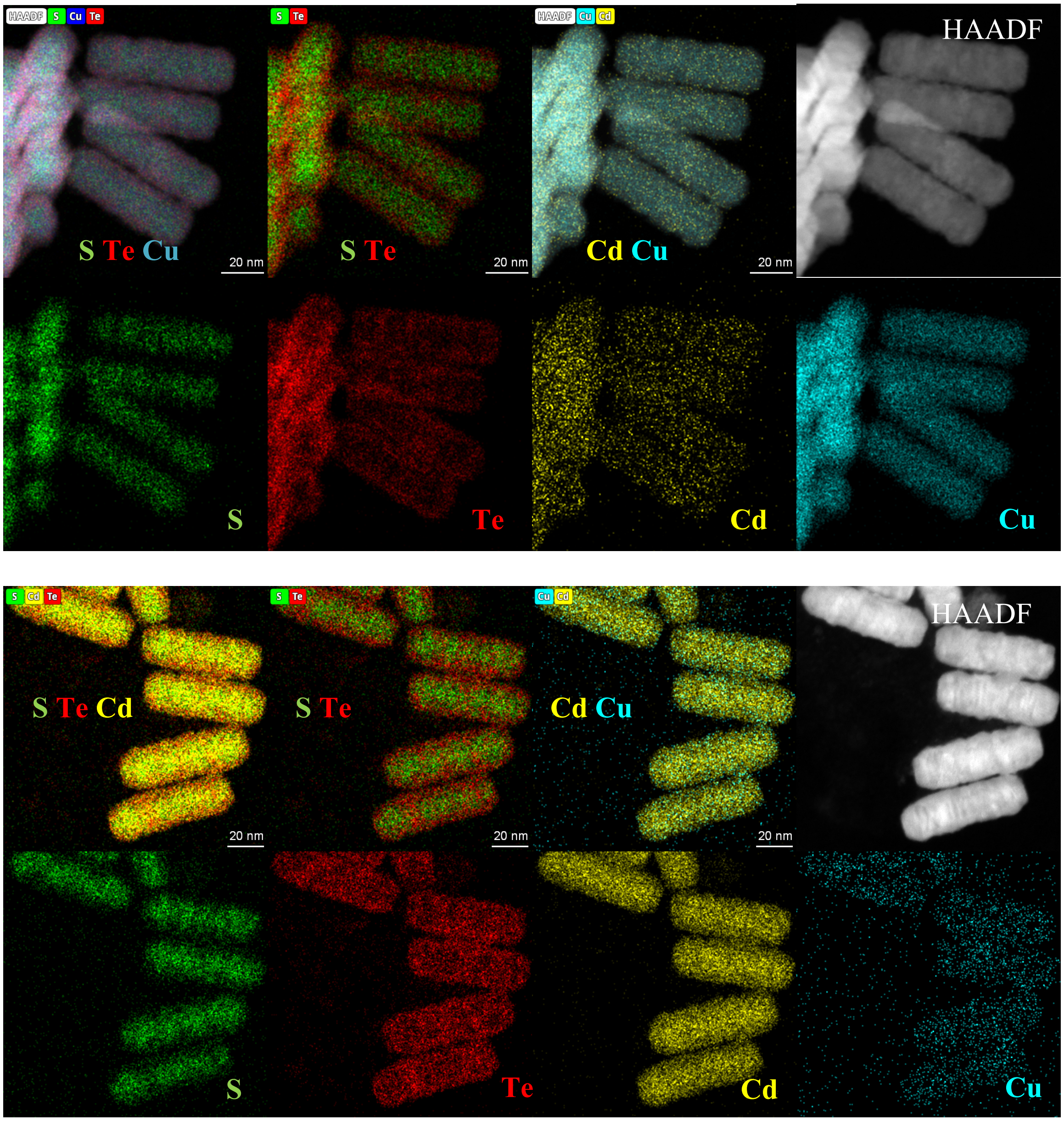

Figure S3. Individual STEM-EDS maps of Te@230 °C particles before (top) and after (bottom) Cd-exchange at 110 °C to create new wurtzite CdS/CdTe nanoheterostructures. Note that Cd is mapped before exchange so that the background signal is apparent.

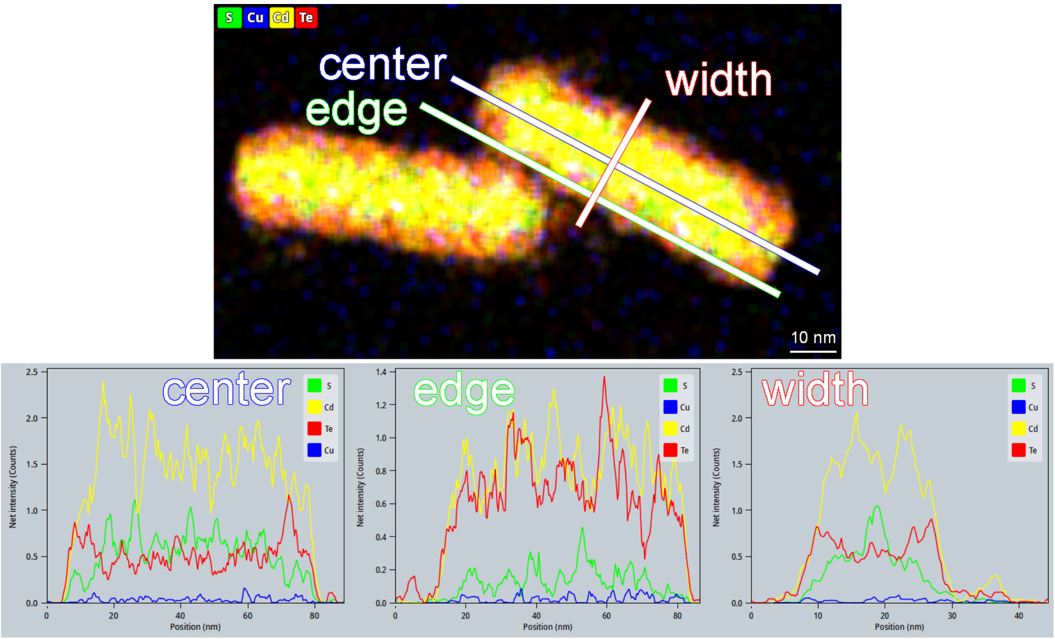

Figure S4. Line scans of a STEM-EDS map of Te@230 °C+Cd@110 °C particles showing the irregular CdS/CdTe core/shell structure. Comparing the edge versus the center shows that Cd and Te are concentrated on the edges, while Te and S are more evenly distributed along the center. This is further supported by a line scan across the width of the particle where Te concentrations spike on the edges and S spikes in the center.

REFERENCES

(1) Garcia-Herrera, L. F.; McAllister, H. P.; Xiong, H.; Wang, H.; Lord, R. W.; O'Boyle, S. K.; Imamovic, A.; Steimle, B. C.; Schaak, R. E.; Plass, K. E. Multistep Regioselectivity and Non-Kirkendall Anion Exchange of Copper Chalcogenide Nanorods. *Chem. Mater.* **2021**, *33* (10), 3841–3850. DOI: 10.1021/acs.chemmater.1c01107.

(2) Espinosa, A. R.; Novak, M.; Luo, Q.; Hole, B.; Doligon, C.; Prenza Sosa, K.; Gray, J. L.; Rossi, D. P.; Plass, K. E. Heterostructures of $Cu_{2-x}S/Cu_{2-x}Te$ Plasmonic Semiconductors: Disappearing and Reappearing LSPR with Anion Exchange. *Chem. Commun.* **2022**, *58* (70), 9810–9813. DOI: 10.1039/D2CC01859D.
